# Severe neonatal enterovirus infection in twins with different outcomes: A case report

**DOI:** 10.3389/fped.2023.1181698

**Published:** 2023-09-06

**Authors:** Kelly K. Storm, Daan De Herdt, Karen Couderé, Jaco J. Verweij, VIRO-TypeNed, Leo Torn, Tim Hundscheid, Herbert M. van Wering

**Affiliations:** ^1^ Department of Pediatrics, Amphia Hospital, Breda, Netherlands; ^2^ Division of Neonatology, Department of Neonatal and Pediatric Intensive Care, Sophia Children's Hospital, Erasmus University Medical Center, Rotterdam, Netherlands; ^3^ Department of Pediatrics, Antwerp University Hospital, Edegem, Belgium; ^4^ Microvida, Laboratory of Medical Microbiology and Immunology, Elisabeth-TweeSteden Hospital, Tilburg, Netherlands; ^5^ Department of Neonatology, Amalia Children’s Hospital, Radboud University Medical Center, Nijmegen, Netherlands

**Keywords:** neonatology, enterovirus, sepsis, myocarditis, ECMO, twins

## Abstract

Enteroviruses are among the most common causes of acute viral illness worldwide, and in neonates, the clinical course of these infections is heterogeneous. Severe complications, such as myocarditis, are associated with high mortality rates. In this case report, we present the clinical course of premature twins born at 35 weeks of gestational age, suffering from a severe neonatal enterovirus infection with cardiac involvement, which proved fatal in one of the twins. This course led to prompt identification in the other twin and facilitated timely transfer to a neonatal intensive care unit with neonatal hemodynamic expertise, and facilitated the timely transfer to a neonatal intensive care nit with hemodynamic expertise and immediate availability of AZCMO would it have been indicated. Early supportive therapy in the other twin contributed to a positive outcome. Therefore, we emphasize the importance of early recognition in averting adverse consequences. As a recommendation, we propose routine screening of enterovirus in viral panels for febrile newborns.

## 1. Introduction

Enteroviruses (EV) are a major cause of acute viral illness worldwide (1), typically resulting in mild symptoms that are usually self-limited in children and rarely lead to long-term complications. In newborns younger than 28 days (neonates), the clinical course is rather heterogeneous, ranging from asymptomatic infection to severe, life-threatening disease with manifestations such as sepsis, myocarditis, hepatitis, coagulopathy, pneumonia, and meningoencephalitis (2–6). Particularly, myocarditis and hepatitis with coagulopathy are associated with high mortality rates (2, 5). The majority of severe neonatal EV infections are caused by coxsackievirus B (CVB) (mainly CVB1 and CVB3) and echoviruses (mainly E11) (2, 7). We present the case of premature twins with severe CVB1 myocarditis, in order to raise awareness of the potentially serious course of a common pathogen and to demonstrate that, when properly anticipated, we might improve the outcome.

## 2. Case

We describe the case of female dichorionic diamniotic (DCDA) twins, born in November 2021 in the Netherlands after 35 completed weeks of gestation, delivered by secondary cesarean section because of preterm premature rupture of membranes (PPROM) while both twins were in breech presentation. Twin A had a birth weight of 2,050 g (with a growth percentile of 9), and twin B weighed 2,330 g (with a growth percentile of 38) at birth. Both had Apgar scores of 9 and 10 at 1 and 5 min, respectively.

In the last few weeks before giving birth, the mother suffered a non-specific illness with coughing and diarrhea without fever. A few hours postpartum, she developed fever, and her blood results revealed signs of inflammation with leukocytosis, lymphopenia, and neutrophilia. Given the suspicion of infection, the mother was treated with intravenous antibiotics (amoxicillin–clavulanic acid) after collection of blood and vaginal cultures. An infection with SARS-CoV-2, influenza A and B virus, respiratory syncytial virus (RSV), human metapneumovirus (hMPV), parainfluenza, and rhinovirus were excluded by real-time reverse transcriptase–polymerase chain reaction (RT–PCR) on a nasopharyngeal swab. Because of a favorable diagnosis in both neonates with no signs of infection, the practice of watchful waiting was adopted.

### 2.1. Disease course of twin A

On day 4 of life, twin A became lethargic and less eager to drink and developed fever up to 38.2°C. Blood results showed leukopenia (5.1 × 10E9/L) and mild thrombocytopenia (117 × 10E9/L), and no blood culture nor CRP (C-reactive protein) was taken because of a lack of material. Antibiotic treatment with amoxicillin and cefotaxime was started because of a suspicion of a late-onset sepsis. The result of the multiplex in-house PCR test for a panel of respiratory tract viruses, such as SARS-CoV-2, rhinovirus, hMPV, RSV, influenza A and B virus, and parainfluenza virus, was negative. In the following days, she developed the need for respiratory support through high-flow nasal oxygen because of desaturation and apneas. Her temperature kept fluctuating despite treatment with antibiotics, lethargy and difficulty in drinking persisted, and thrombocytopenia worsened to a minimum of 11 × 10^9^/L on day 6, with a peak CRP of 9 mg/L. At this moment, her stool was collected for viral diagnostics, such as norovirus, sapovirus, adenovirus, parechovirus, and EV, and her urine was examined for the possible presence of cytomegalovirus (CMV). Echo cerebrum did not show any signs of a thrombus or bleeding. Because of the risk of spontaneous bleeding, a platelet transfusion was given on day 6 with an adequate post-transfusion thrombocyte increment to 68 but with ongoing platelet consumption. Because of paired QRS complexes on the cardiac monitor, an electrocardiogram (ECG) was taken, but it did not show any abnormalities.

On day 7, the neonate's clinical condition deteriorated acutely, with circulatory insufficiency expressing as a marbled skin, prolonged capillary refill of 3–4 s, and alternating brady- and tachyarrhythmias. This quickly resulted in cardiac arrest with the need for cardiopulmonary resuscitation (CPR). Point-of-care ultrasound (POCUS) did not show any intracranial or abdominal bleeding, which was initially suspected, given the thrombocytopenic status of the patient. Echocardiography of the heart showed a significantly reduced cardiac output without signs of congenital heart disease. No pericardial effusion and no dilatation of the heart were reported. After 1 h of CPR, using adrenalin following neonatal CPR guidelines and dobutamine to enhance cardiac contractility, the treatment was discontinued because no recovery of spontaneous circulation and no option for extracorporeal membrane oxygenation (ECMO) were observed. The patient died on the seventh day of life.

**Figure 1:**
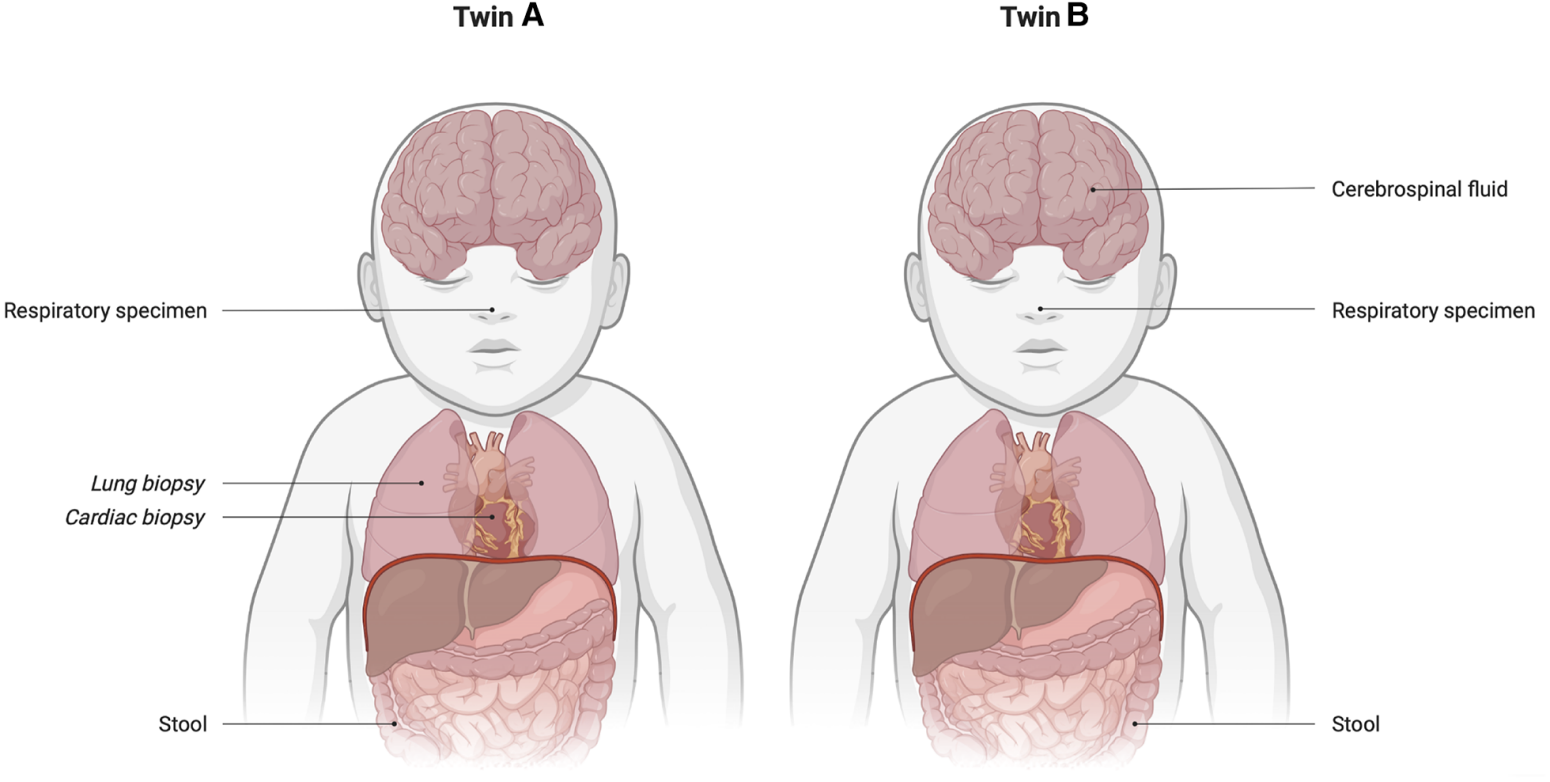
Overview of (positively) tested samples on enterovirus for each twin. Cardiac and lung biopsies were obtained during postmortem examination.

Around the time of death, we were reported that an EV was detected by RT–PCR in her stool. This finding led us to infer that the presumed cause of death was EV myocarditis. Subsequently, EV was also identified in the respiratory specimen collected earlier, with further analysis revealing its type as CVB1. Postmortem examination showed multiple macrophages and CD3+ lymphocytes in the heart, lungs, and adrenal glands. A biopsy of the heart and lungs was positive for EV. These findings definitively confirmed the diagnosis of EV myocarditis. Figure 1 provides an overview of all tested samples for EV, all of which tested positive.

### 2.2. Disease course of twin B

Twin B was still asymptomatic on day 4. However, because of suspicion of a late-onset sepsis in her sister, a blood culture was taken, and antibiotic treatment with amoxicillin and cefotaxime was started. Her CRP level repeatedly remained below 1 mg/L, and her blood culture remained negative. On day 7, abdominal distension was found during clinical observation. An x-ray of the abdomen showed no abnormalities. After the fatal circulatory failure of her sister, an echocardiogram was performed, which revealed a structurally normal heart with good cardiac function and contractibility. On the same night, she started showing increasing respiratory distress. Blood results revealed decreasing thrombocytes of 134 × 10^9^/L with an increased N-terminal pro-B-type natriuretic peptide (NT-proBNP) of 3,793 pmol/L and cardiac troponin I of 2,350 ng/L. Her CRP level was less than 1 mg/L. At risk of also having an EV infection with cardiac involvement, and with the clinical course of twin A in mind, she was transferred to a neonatal intensive care unit (NICU) of a tertiary hospital that offered ECMO.

In the NICU, the neonate's cardiac function worsened. EV was found in her stool, respiratory specimen, and cerebrospinal fluid (CSF) (Figure 1), later also typed as CVB1. An ECG showed repolarization abnormalities indicating myocardial damage. Intravenous immunoglobulins (IVIG) in a high dose of 2 g/kg were administered over 48 h as EV myocarditis was suspected. Left ventricle function worsened with the development of pulmonary hypertension, for which milrinone was given. Because of a low flow state, carbasalate calcium was administered as anticoagulation. She was intubated to reduce her energy expenditure. Because of the clinical course in twin B, the same enterovirus subtype, and echocardiographic findings, a diagnosis of EV myocarditis was made. Cardiac biopsy was not performed, given the lack of clinical consequences and risk of the procedure.

**Figure 2:**
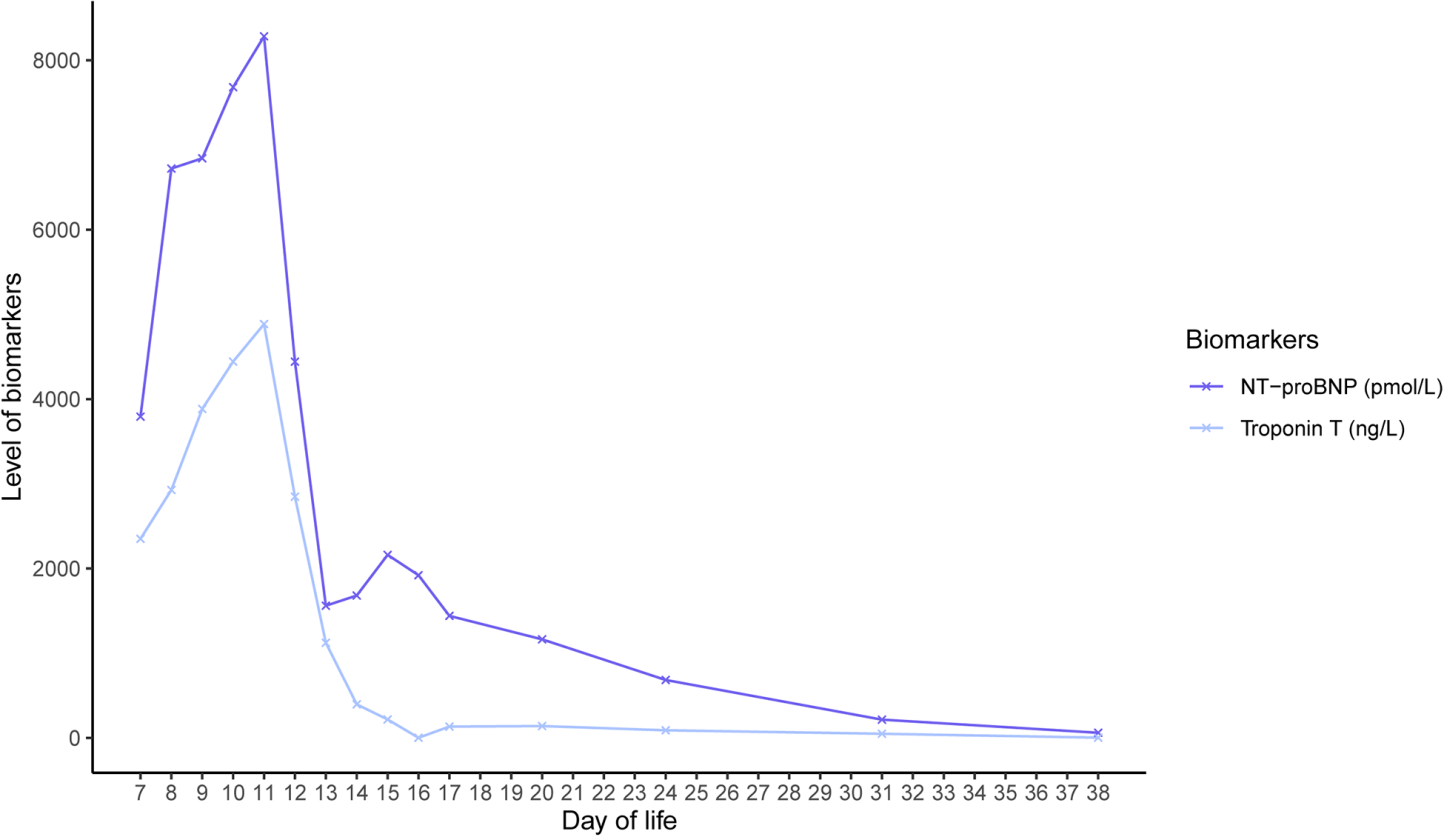
Course of cardiac biomarkers NT-proBNP and troponin T in twin B.

After the interventions, there was a decrease in cardiac enzymes and an improvement in left ventricle function (Figure 2). After 3 days, she was extubated. Enalapril and spironolactone were prescribed as maintenance drugs, and milrinone was discontinued. After 39 days of hospitalization, of which 20 days were spent on the NICU, twin B was discharged home.

Follow-up in outpatient clinics took place, where the patient's general wellbeing, development, and cardiac function were monitored. She fully recovered from her severe EV myocarditis, with normal heart function and no residual damage at 12 months of follow-up without maintenance drugs.

## 3. Discussion

**Figure 3:**
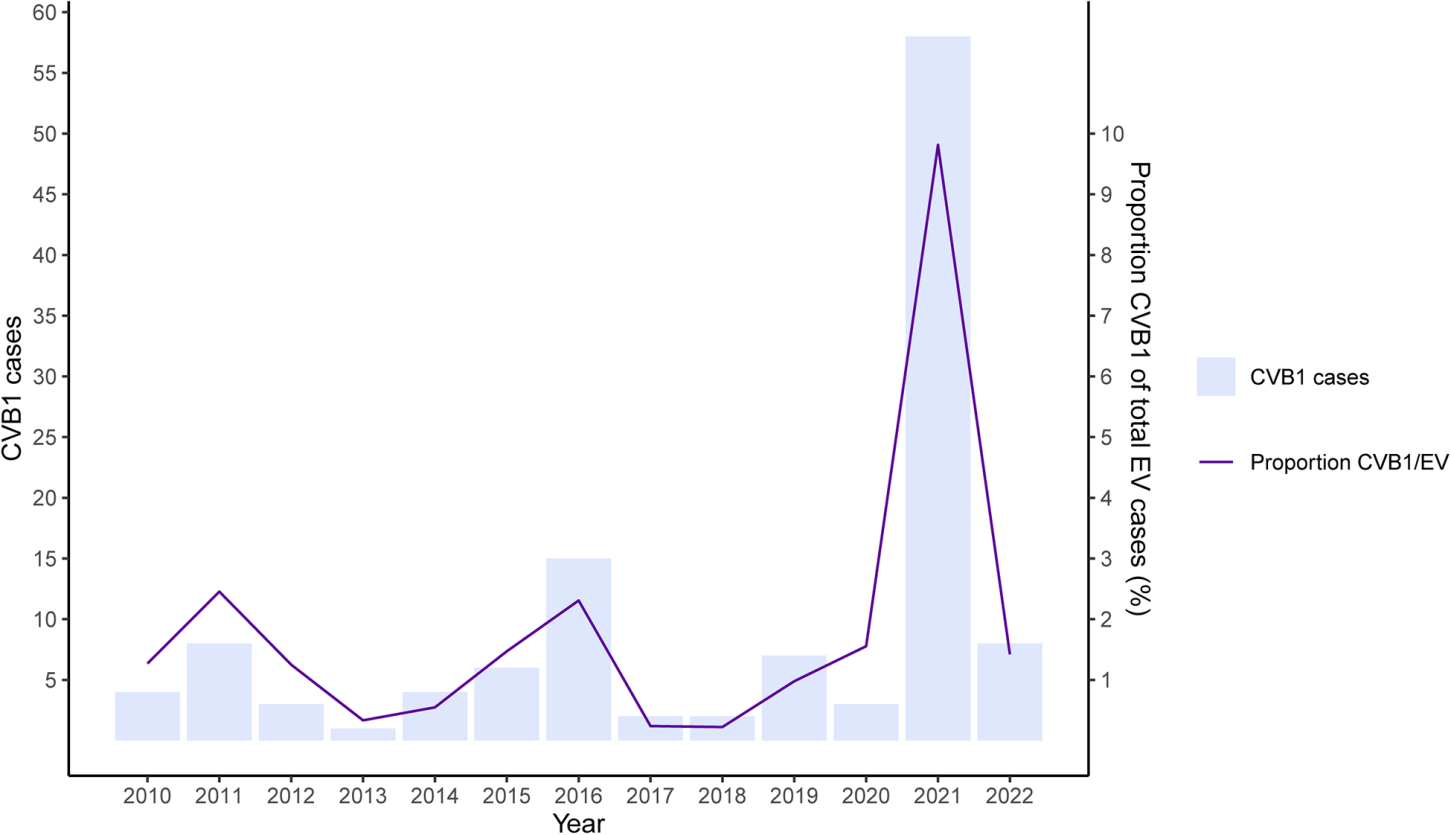
Occurrence of CVB1 in the Netherlands. The figure gives the absolute number of CVB1 infections (bars) and proportion of CVB1 of total EV infections (line) between 2010 and 2022.

According to the Dutch National Surveillance system (VIRO-TypeNed), CVB1 experiences upsurges every 5 years, with notable increases in the years 2011, 2016, and 2021 (Figure 3). The number of CVB1 cases in 2021 was the highest since the last two decades, and it presented as one of the predominant types. EV circulation is primarily driven by population immunity (8), which might have been affected by reduced transmission during the COVID-19 pandemic, potentially contributing to an exceptional surge in cases in the year 2021 (9). Other neonatal myocarditis cases caused by EV infections were reported in the Netherlands in the year 2021 by the Signaling Consultation of the National Institute for Public Health and the Environment (RIVM).

Although no type is strictly limited to a particular disease or vice versa, there seems to be some association between certain EV types and disease presentation and severity, such as the association between CVB types and myocarditis or severe neonatal infections (2, 10). In the year 2007, a notable outbreak in the United States reported five fatal cases of neonatal sepsis, all attributed to CVB1 (11).

Nevertheless, the precise disease burden of CVB types and myocarditis remains unknown because of the lack of standardized data collection. To address this gap, clinical and virological monitoring and reporting are essential to increase awareness among clinicians, particularly with regard to the potential detrimental course of CVB-associated neonatal sepsis (12), as evident in the case of twin A.

The European Non-Polio Enterovirus Network (ENPEN) (13) has been established to study and monitor these data, including circulating types and (limited) clinical data, within the European region. In our case, while we conducted typing based on the sequencing of viral envelope protein 1 (VP1), whole-genome sequencing of the CVB1 strains was not performed.

Neonatal EV infection often presents with non-specific symptoms, such as temperature abnormalities, rash, and poor feeding. In addition, a range of heterogeneous symptoms have been described, such as respiratory disease, lethargy, jaundice, circulatory failure or shock, arrhythmias, thrombocytopenia, poor perfusion, irritability, hypotonia, and diarrhea (2). Both twins presented with several of these symptoms, which are hard to distinguish from bacterial or other viral infection (1). Lv et al. identified an abnormal CSF test, thrombocytopenia, highest temperature of >38.35°C, duration of fever at >3.25 days, and a negative bacterial culture as significant predictors of having an EV infection (14). They reported an overall EV PCR positivity of 39.2% in febrile neonates, suggesting routine EV diagnostics for all febrile cases. Other studies also support this, reporting EV-positive PCR tests on blood and/or CSF in neonates with fever, sepsis-like disease, or suspected meningitis or encephalitis, with the rate of incidence ranging from 11.6% to 60.2% (15–17).

Zhang et al. conducted a systematic review on severe enterovirus infections in 237 neonates (2). Most cases (70.5%) exhibited symptoms within the first 7 days of life, and 29.5% were associated with maternal disease before delivery (2). Vertical transmission with early disease onset poses a significant risk factor for a severe infection because of the absence of serotype-specific transplacentally acquired neutralizing antibodies (18). Prematurity, male sex, positive viral culture, and evidence of severe hepatitis and multisystem disease were identified as additional risk factors (5, 7, 18–20).

In our case, both twins manifested symptoms within the initial 7 days of life, strongly indicating perinatal transmission. This theory gains further support from the mother's illness prior to birth, although EV testing was not performed because of parental refusal. Notably, the twins were premature, and EV RT–PCR yielded positive results for both infants. Consequently, they presented with a confluence of multiple risk factors that significantly predisposed them to a potentially severe course of infection. No further research into immunodeficiencies was conducted.

Various severe manifestations of neonatal EV infection have been described, including hepatitis or coagulopathy, myocarditis, and meningoencephalitis (2). Mortality rates for these presentations range from 11.5% to 38.5%, with the highest observed in myocarditis cases (2, 21). Among EV myocarditis patients, 44.3% had CVB infection, primarily CVB1 and CVB3 (2). Clinical signs may include respiratory symptoms (27.3%), arrhythmias (30.7%), circulatory failure or shock (39.8%), and poor perfusion (12.5%). The case of twin A exemplifies the rapid deterioration of ventricle function attributable to EV myocarditis, leading to sudden cardiovascular collapse and contributing to high mortality rates (2, 22). Therefore, early recognition and awareness of this severe cardiac manifestation are crucial.

ENPEN's recent recommendation advised performing EV RT–PCR on CSF, blood, stool, and respiratory samples promptly after symptom onset. Sampling multiple sites is crucial as viral loads can be higher in blood than CSF, and detection remains prolonged in stool and respiratory secretions (12). When stool is unavailable, rectal swabs, though less sensitive, offer a valuable alternative (23). In the case presented, enterovirus diagnostics was not performed on the initial respiratory sample, causing a delay in diagnosis.

Treatment for neonatal myocarditis is primarily supportive, but the optimal strategy lacks conclusive evidence. Reducing oxygen demand is essential, often achieved through intubation and ventilation. Milrinone or dobutamine can be employed for afterload reduction. Inotropic agents increase the contractility of the heart but may also cause an increase of myocardial oxygen consumption (24).

IVIG, containing plasma from at least 1,000 blood donors, contains antibodies against common circulating enterovirus types (25). Apart from its recognized immunomodulatory effects, IVIG may also exhibit a direct antiviral effect. However, literature on IVIG as a treatment for neonatal EV infections is scarce. Abzug (5) explored its potential positive impact in 16 neonates with EV infection. Among the treated group, nine neonates received IVIG at a dosage of 750 mg/kg, resulting in a mild increase in neutralizing antibodies. However, it did not significantly reduce the viral load in blood and urine or impact major clinical outcomes. Another study by Yen et al. demonstrated that early IVIG administration (within 3 days after the symptoms started) correlated with lower mortality than late IVIG (26). Thus, prompt EV suspicion in severe neonatal sepsis could be crucial to initiate IVIG treatment as early as possible. Twin A's cardiac failure resulting from an EV infection raised concerns about twin B's disease severity, leading to early IVIG treatment, potentially influencing her outcome positively. No antivirals were administered. Several antivirals and host factor-target inhibitors have been under development and assessed in clinical trials (27, 28). However, currently, no antivirals are available for routine clinical practice.

In patients with myocarditis and cardiac failure unresponsive to conventional therapy, ECMO is a valuable option and should be considered as a bridge to recovery. Unfortunately, ECMO was not available in the center where twin A collapsed, but awareness and early recognition facilitated timely transfer to an NICU with hemodynamic expertise, where ECMO could have been promptly available if needed. The optimal timing for initiating ECMO in neonatal myocarditis cases lacks clear guidance (29). Ford et al. (30) studied over 4,000 neonates with heart disease and found that severe acidosis and increased duration of mechanical ventilation prior to ECMO initiation were associated with increased mortality. These findings suggest that earlier initiation of ECMO might be beneficial for reducing mortality (29, 30). However, starting ECMO too early in the disease course is associated with severe morbidity.

In a study by Cortina et al. (22), the combined survival rate for neonatal EV myocarditis cases requiring ECMO was 35.7%. Despite ECMO's high risk of complications and mortality (29), favorable long-term outcome with complete cardiac recovery is possible.

Literature on the long-term cardiac prognosis for survivors of enterovirus myocarditis in the neonatal period is scarce. Freund et al. discussed seven cases and their follow-up (21), revealing two fatalities within the first 2 months of age due to circulatory failure. Among the five survivors, none fully recovered. All developed dilated cardiomyopathy with mild or severe aneurysm formation within the left ventricle, and their follow-up period ranged from 13 months to 15.7 years. To augment their findings, the authors conducted a literature review encompassing 28 cases. The overall mortality rate was 35%, with the majority of the survivors (66%) experiencing sequelae, independent of gestational age. Reported sequelae included residual myocardial dysfunction, chronic calcific myocarditis with chronic heart failure and dysrhythmias, and ventricular aneurysm. Interestingly, only 23% (8/35) of the previously reported infants achieved full recovery such as our case of twin B, who not only survived but also fully recovered without any residual damage or need for maintenance therapy at 1 year follow-up.

## 4. Conclusion

In this particular case, twin A's cardiac failure resulting from enterovirus infection raised concern about the potential severity of the disease in twin B. Consequently, twin B was transferred to an ECMO center and received IVIG treatment and cardiorespiratory support at an early stage.

The overall tendency toward enterovirus infection is negligent, given the overall favorable course in children, but cardiac involvement is associated with high morbidity and mortality (2). Supportive therapy with IVIG, energy management with elective intubation, and cardiovascular support might be beneficial. If supportive therapy is unsuccessful, ECMO can serve as a bridge to recovery if promptly initiated, underscoring the importance of early recognition. Because clinical signs of severe disease usually appear late, routine screening of enterovirus in viral panels from multiple compartments for febrile newborns is suggested. Additional research is required to establish a standard diagnostic workup for children with enteroviral infections in order to differentiate between a benign, self-limiting infection and a potentially severe disease. Awareness of severe disease and therefore early transfer to an ECMO center might lead to better outcomes.

## Data Availability

The original contributions presented in the study are included in the article/supplementary material, further inquiries can be directed to the corresponding author.
